# Context-dependent rhythmicity in chimpanzee displays

**DOI:** 10.1098/rspb.2024.2200

**Published:** 2024-12-04

**Authors:** B. J. R. van der Vleuten, V. A. Hovenkamp, J. M. Varkevisser, M. J. Spierings

**Affiliations:** ^1^ Institute of Biology Leiden, Leiden University, Leiden 2333 BE, The Netherlands; ^2^ Leiden Institute for Brain and Cognition, Leiden University, Leiden 2333 AK, The Netherlands; ^3^ Department of Behavioral and Cognitive Biology, Vienna University, Vienna 1030, Austria

**Keywords:** chimpanzee, isochrony, display behaviour, rhythmic social behaviour, comparative cognition, music evolution

## Abstract

Rhythm is an important component of human language and music production. Rhythms such as isochrony (intervals spaced equally in time) are also present in vocalizations of certain non-human species, including several birds and mammals. This study aimed to identify rhythmic patterns with music-based methods within the display behaviour of chimpanzees (*Pan troglodytes*), humans’ closest living relatives. Behavioural observations were conducted on individuals from two zoo-housed colonies. We found isochronous rhythms in vocal (e.g. pants, grunts and hoots) as well as in motoric (e.g. swaying and stomping) behavioural sequences. Among individuals, variation was found in the duration between onsets of behavioural elements, resulting in individual-specific tempi. Despite this variation in individual tempi, display sequences were consistently structured with stable, isochronous rhythms. Overall, directed displays targeted at specific individuals were less isochronous than undirected displays. The presence of rhythmic patterns across two independent colonies of chimpanzees suggests that underlying mechanisms for rhythm production may be shared between humans and non-human primates. This shared mechanism indicates that the cognitive requirements for rhythm production potentially preceded human music and language evolution.

## 1. Introduction

Rhythm plays a key role in the production and perception of language and music in humans. The foundation of most rhythms present in language and music is isochrony: Every consecutive interval between actions has approximately the same duration [1]. This form of consistency even appears in basal physiological processes, such as a beating heart and respiration [2,3]. In the past few decades, a stronger focus on the comparative approach has led to increasing numbers of non-human animals being studied for their rhythmic capacities. Research in the field of comparative behavioural studies has shown that traits for perception and production of simple (e.g. isochronous) rhythms seem to be universally present in a broad range of species [4–6]. More elaborate rhythmic capabilities—for instance, entrainment to an external sound pulse—are likely exclusive to humans and relatively few other species [7–10]. This universal base for rhythm perception and production, with the exception of advanced skills in a few species, points toward a gradual process of small cognitive and motoric changes over time that eventually evolved into the elaborate rhythmic capacities used in language and music [11]. However, to understand the cognitive underpinnings of rhythmicity and to form evolutionary theories of rhythmicity, additional empirical data from a comparative perspective are necessary.

Rhythm seems to permeate behaviours across species; nevertheless, there is a difference between rhythm present in intentional, goal-oriented behaviours rooted in deeper cognitive processes and behaviours that are not actively controlled (e.g. an isochronous heartbeat). To quantify rhythm in intentional animal behaviour, a generalized music-based method was used in a study by Roeske *et al*. [12], who researched the similarities between birdsong and human music. Similarly to human music, the song of thrush nightingales (*Luscinia luscinia*) and zebra finches (*Taeniopygia guttata*) has an underlying, isochronous structure, with additional categorical rhythms, such as small-integer ratios (SIR) of 1 : 2, where consequent intervals have twice the duration of prior intervals. Likewise, using this or a highly similar method, spontaneously produced, rhythmic vocalizations have been quantified in a few mammals: bats (*Saccopteryx bilineata*) [13], seal pups (*Phoca vitulina*) [14], hyraxes (*Procavia capensis*) [15], lemurs (*Indri indri*) [16], gibbons (*Hylobates lar* [17], *Nomascus gabriellae*, *Nomascus leucogenys* and *Nomascus siki* [18]) and orangutans (*Pongo pygmaeus wurmbii*) [19]. These primates (lemurs, gibbons and orangutans) show isochrony or other categorical rhythms in their vocalizations, further indicating that the evolutionary origins of these traits emerged before the lineage split between humans and other primates. Moreover, recent efforts to study isochrony in motoric behaviour, rather than vocalizations, found a significant tendency toward isochrony in the grass-plucking movements of wild geladas (*Theropithecus gelada*) [20]. These promising results show that these methods are suitable for quantifying rhythmic behaviour. Similar approaches will help gather more data, enabling comparisons between species and advancing our knowledge of the evolutionary trajectory of rhythm production.

Currently, rhythm production and perception still need to be assessed across the majority of the primate species, although earlier research has suggested temporal consistency within primate social behaviour. Regarding humans’ closest living relatives, chimpanzees (*Pan troglodytes*) drum on the buttresses of trees with a steady cadence in the wild [21]. This behaviour is commonly used for locating and coordinating subgroups over longer distances during moving events [21], with individual- and group-specific variation to ensure clear recognition within and between groups [22]. The chimpanzees select buttressing tree roots with specific sound propagating features, indicating that it is a non-random, intentional behaviour [23]. These drum sessions are often accompanied by pant-hoot call sequences, which have similar inter-individual and group-specific variations to the buttress drumming behaviour [22,24,25]. Besides regularity in long-distance communication, there has been a recorded instance of a zoo-housed chimpanzee drumming on a barrel within its enclosure in a constant pattern, with occasional changes in tempo [26]. Even though such observations as described in this anecdotal report have not frequently been reported ever since, they do further enhance the likelihood of rhythmic patterns, similar to those found in language and music, being present in chimpanzee behaviour.

This study focuses on rhythm in the structured display behaviour of zoo-housed chimpanzees. In display behaviour, (mostly) male chimpanzees use an elaborate repertoire of vocalizations and motoric movements [27,28] to establish dominance, intimidate hierarchical challengers, resolve conflict, attract a mate or to play [27,29–31]. The sequences of recurring movements, such as drumming, chest-beating and swaying, are accompanied by frequent vocalizations, such as pants, hoots, grunts and screams. These multimodally produced behavioural sequences form the ideal opportunity for studying rhythm in these great apes. Display behaviour can be either directed, with an optical and bodily orientation toward the target individual(s) [29], or undirected, with no immediate target, which is rather a show of strength, endurance or frustration toward the entire group [32]. When rhythm can be deducted from display behaviour sequences, it is also possible to assess whether this rhythmicity remains consistent or varies across different contexts.

We measured rhythmicity in chimpanzee display behaviour by extracting the inter-onset intervals (the intervals between the starts of two consecutive vocalizations or movements) and ratios between different behavioural elements, a well-established method for rhythmic analyses [12,33]. We have taken into consideration inter-individual patterns and tempo variations between the displays of each individual. Besides that, other factors that might influence rhythm production, such as the mode of production (vocal or motoric) and individual variation, were examined. Quantifying rhythm and understanding which factors influence rhythm in chimpanzee behaviour provide a greater understanding of whether certain aspects of rhythm perception and production are universal across species. Adopting this generalized method in comparative rhythm research allows us to form evolutionary theories of rhythmicity, which has been rudimentary to the evolution of human language and music.

## 2. Material and methods

### (a) Animals and recordings

The data were collected by observing the chimpanzees (*P. troglodytes*) of two colonies housed in Beekse Bergen (Hilvarenbeek, The Netherlands) and Burgers’ Zoo (Arnhem, The Netherlands). In total, 43 individuals were observed, 26 individuals resided in Beekse Bergen and 17 individuals in Burgers’ Zoo. Though the age ranges of the two colonies are similar, the individuals housed in Burgers’ Zoo were, on average, 12 years older. Furthermore, a substantial number of the individuals housed in Beekse Bergen have been part of a control group for medical research at the Biomedical Primate Research Centre (BPRC, Rijswijk, The Netherlands). All other observed individuals were born and raised in their current, or other, zoos.

Each colony was divided into two separate groups, which were within audible distance of each other. The groups occupied enclosures with inside and outside habitats; however, observations were only made outside to allow accurate recording of the displays. All behaviours were recorded using JVC Everio GZ-R415 video cameras, a Sennheiser directional microphone and a Marantz Professional PMD661 audio recorder during all-occurrence sampling sessions that lasted up to 5 h. The lengths of sessions depended on the time spent outside by the individuals. In total, nearly 90 h of recordings were collected, spread over 35 days (April–May in Beekse Bergen and September–November in Burgers’ Zoo). Only vocalizations that were clearly attributable to a specific individual were included in the study. This resulted in 132 displays (included *vocal* = 73 and *motoric* = 110) performed by 29 individuals. Additional information regarding group composition and individuals’ contribution to displays can be found in the electronic supplementary material, S1 and S2.

### (b) Visual and acoustic analysis

Before analysing, raw videos and audio recordings were synchronized, and individual display sequences were then isolated from the continuous recordings with video-editing software [34,35]. The isolated WAV audio files were annotated with Praat 6.3.10 [36] (*vocal*) and MP4 video files with BORIS 7.13.6 [37] (*motoric*). The onsets and offsets of all calls (by call types: grunts, hoots, screams, barks and pants) or motoric actions (see Ethogram; electronic supplementary material, S3) were noted within the display sequences. The onset times were then used to calculate the inter-onset intervals (*t*_*k*_) between consecutive behavioural elements (i.e. vocalizations and motoric movements). To assess the variability of these intervals, the coefficient of variation (ratio of the standard deviation to the mean of the inter-onset intervals in a sequence) was computed for each behavioural sequence. Because of the small number of intervals in some of the sequences, we used the formula for an unbiased estimator [33,38]. A lower coefficient of variation indicates more regular intervals in a sequence. To quantify rhythmic patterns within the behavioural sequences, the ratio (*r*_*k*_) between consecutive inter-onset intervals was computed by $r_{k}=\frac{t_{k}}{t_{k}+t_{k+1}}$ . Here, the calculated interval ratios translate to numeric values between 0 and 1, where an interval ratio of 1 : 1, *r*_*k*_ = 0.500, marks isochrony. Besides isochrony, other categorical rhythms with ratios of 1 : 3 (*r*_*k*_ = 0.250), 1 : 2 (*r*_*k*_ = 0.333), 2 : 1 (*r*_*k*_ = 0.667) and 3 : 1 (*r*_*k*_ = 0.750), known as SIR, were analysed. Interval ratios were considered integer (rhythmic) when they fell within the pre-set boundaries of the categorical rhythms [12]. Off-integer (arrhythmic) interval ratios, grouped outside the rhythmic ranges, form the counterparts of the integer interval ratios.

### (c) Statistical analysis

All statistical analyses were performed in R v. 4.2.2 [39]. To quantify rhythmicity in the display behaviour, we adopted the methodology previously proposed by Roeske *et al*. [12], Anichini *et al*. [14] and De Gregorio *et al*. [16]. First, the calculated interval ratios between consecutive elements were grouped into bins of the categorical rhythms. Here, only individuals with at least 50 interval ratios across the entire dataset were included to make a proper comparison. The modes of production (vocal/motoric) were also separately analysed, including only individuals with at least 25 interval ratios in the corresponding mode (electronic supplementary material, S1 and S2). Following this, the interval ratios of the preselected individuals were visualized in density plots (data visualization; R packages *ggplot2* [40], *gridExtra* [41], *reshape* [42] and *patchwork* [43]). To assess whether the observed distribution could be a product of chance, we compared it with a generated uniform distribution of interval ratios. Following Anichini *et al*. [14] and De Gregorio *et al*. [16], we randomly generated two values uniformly distributed between the minimum and maximum of the observed interval range. These values were used to calculate a ratio. By repeating this 100 000 times, we produced a simulated uniform distribution of ratios [14]. This simulates a null ratio distribution without rhythmic categories because each value within the defined range had an equal probability of being selected [16]. For this comparison, an asymptotic two-sample Kolmogorov–Smirnov test was used to determine the likelihood of the observed interval ratios originating from the referential uniform distribution [14].

Furthermore, for each individual, counts of interval ratios within integer and off-integer categories were divided by the categorical bin size to normalize the variable bin sizes between categories. Then, the density of each categorical integer rhythm (i.e. isochrony or SIR) was compared with their neighbouring off-integer counterparts using paired Wilcoxon signed-rank tests.

Next, the average interval duration per display for each individual was calculated and plotted to visualize the inter-individual variation of temporal properties in display sequences. For this comparison between individuals, only those with at least four performed displays were selected from the previously selected group. This prevents biased outcomes where the appearance of high consistency in individuals with fewer displays is merely the result of having fewer intervals.

The influence of zoo, mode of production and directedness of the display on the regularity of the inter-onset intervals was assessed with a linear mixed model (LMM; R package *lme4* [44]; function *lmer* [45]). The response variable in this model was the unbiased coefficient of variation of the different behavioural sequences, and the fixed effects were the housing zoo of the focal individuals (Beekse Bergen or Burgers’ Zoo), the mode of production (i. e. vocal or motoric displays) and directedness of the display (directed or undirected). The displaying individual was added as a random effect factor. Moreover, the influence of these factors on the observed rhythmic patterns was assessed with a generalized linear mixed model (GLMM; R package *lme4* [44]; function *glmer* [45]). In this model, the response variable was the individual counts of isochronous interval ratios, or isochrony rate, which was related to the model via a binomial link function (1 = isochronous ratio and 0 = non-isochronous ratio). The model incorporated the corresponding colony of the focal individuals, mode of production and directedness of the display as fixed effects. Random effect factors were the displaying individual and the specific display sequence (i.e. individual-ID and display-ID). Other factors, such as demography (age and sex), intergroup variation (within zoo colony) and behaviour type (call types/ motoric behaviour categories), did not improve the fit of the model (model assessment; R packages *performance* [46], *DHARMa* [47]) and were, thus, not included (electronic supplementary material, S8).

Finally, *p* values were corrected for multiple testing, using the Benjamini–Hochberg procedure [48], and their significance was tested against *α* = 0.05.

## 3. Results

### (a) Quantification of rhythm

**Figure 1.**
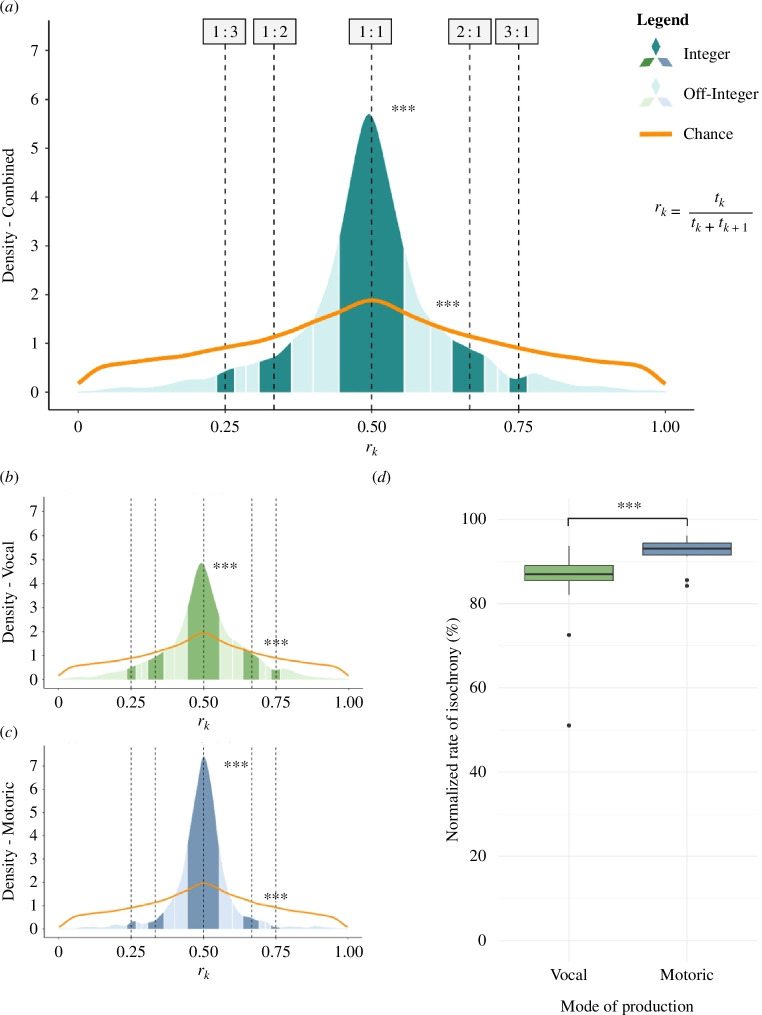
Visualization of the distribution of the calculated interval ratios within the observed display behaviour ((*a*) all behaviours, (*b*) vocal and (*c*) motoric). Integer (darker bins) and off-integer (lighter bins) rhythmic categories are visualized with the categorical rhythms 1 : 3, 1 : 2, 1 : 1 (isochrony, *r*_*k*_ = 0.500), 2 : 1 and 3 : 1, marked with dashed lines. More interval ratios fall within the integer rhythmic boundaries than in the corresponding off-integer bins, and the interval ratios are more likely to be isochronous compared with all other rhythmic categories combined (for (*a*), (*b*) and (*c*)). The high rates of isochrony in the dataset differ significantly from what would be expected by chance (as can be seen from the difference between the observed data and the simulated uniform distribution (orange line; see *methods*)). The motoric elements within the display generally had a higher rate of isochrony than the vocal elements, as depicted in the boxplot, with the rate of isochronous interval ratios (percentage) normalized with bin size (*d*).

First, the density of the interval ratios was plotted to visualize rhythmic patterning (figure 1*a*). The observed interval ratios of the display behaviour result in a distinct peak at a numerical ratio of 0.5, which marks isochrony. Determined with a Kolmogorov–Smirnov test, the observed ratios differed significantly from the distribution that would have been expected by chance (asymptotic two-sample Kolmogorov–Smirnov test; *D* = 0.08, *p* < 0.001). Ultimately, this shows that the observed distribution of ratios is not a product of chance.

To compare the normalized counts of ratios in the different categorical rhythms, Wilcoxon signed-rank exact tests were used because these were not normally distributed (Shapiro–Wilk normality test; *W* = 0.96, *p* < 0.001). The interval ratios of the displays fell significantly more within the bounds of isochrony than in the off-integer bins adjacent to isochrony (Wilcoxon signed-rank exact test; 1 : 1 ratio; integer versus off-integer: *V* = 78, *p* = 0.001). For the other categorical rhythms, only the interval ratios within the 1 : 3 ratio were significantly higher than the off-integer bins adjacent to this category (Wilcoxon signed-rank exact test; 1 : 3 ratio; integer versus off-integer: *V* = 72, *p* = 0.012; electronic supplementary material, S5). The number of interval ratios within isochrony was significantly higher compared to the neighbouring categorical rhythms (Wilcoxon signed-rank exact test; 1 : 2 versus 1 : 1* V* = 0, *p* = 0.001; 1 : 1 versus 2 : 1: *V* = 78, *p* = 0.001). In fact, most of the interval ratios were concentrated within isochrony because the count of isochronous ratios was higher than all other off-integer or integer ratios combined (Wilcoxon signed-rank exact test; isochrony versus off-isochrony: *V* = 78, *p* = 0.001).

No significant difference was found between the variability of the inter-onset intervals and the rates of isochrony of the two isolated colonies (LMM, coefficient of variation; Beekse Bergen versus Burgers’ Zoo: estimate = 0.048, s.e. = 0.072, *t* = 0.665, *p* = 0.516; GLMM, isochrony rate; Beekse Bergen versus Burgers’ Zoo: estimate = 0.068, s.e. = 0.237, *Z* = 0.287, *p* = 0.774; electronic supplementary material, S4). This suggests that the observed rhythmic patterns remain consistent despite the environmental or genetic differences between the colonies.

### (b) Mode of production

Furthermore, the influence of the mode of production on interval variability (coefficient of variation) and rhythm in the display was evaluated. Interval variability was significantly higher in the vocal sequences than in the motoric sequences (LMM, *coefficient of variation*; motoric versus vocal: estimate = 0.175, s.e. = 0.036, *t* = 4.871, *p* < 0.001; electronic supplementary material, S4). Moreover, a significantly higher rate of isochrony was found within the motoric sequences, compared to the vocal sequences (figure 1*b*,*c*: GLMM, isochrony rate; motoric versus vocal: estimate = −0.892, s.e. = 0.136, *Z* = −6.537, *p* < 0.001; electronic supplementary material, S4). Nevertheless, for both the vocal and motoric sequences, the generated interval ratios were heavily centred around isochrony. Additionally, comparing both modes with their individually generated chance distribution shows that the vocal and motoric ratio density plots significantly differed from what would be expected by chance (asymptotic two-sample Kolmogorov–Smirnov test; vocal: *D* = 0.08, *p* < 0.001; motoric: *D* = 0.16, *p* < 0.001). Relatively more interval ratios were produced in an isochronous rhythm compared with all other categorical rhythms (Wilcoxon signed-rank exact test: isochrony versus off-isochrony; vocal: *V* = 105, *p* < 0.001; motoric: *V* = 66, *p* = 0.002). Similarly, the ratio density was higher within isochrony than in the corresponding off-integer bins (Wilcoxon signed-rank exact test; 1 : 1 ratio; integer versus off-integer; vocal: *V* = 104, *p* < 0.001; motoric: *V* = 66, *p* = 0.002). L’ikewise, there were hardly any significant difference between any of the other categorical rhythms and their specific off-integer opposites (electronic supplementary material, S5).

In either mode of production, there were no statistical differences in the ratio distribution between the different zoos (asymptotic two-sample Kolmogorov–Smirnov test; *Beekse Bergen* versus *Burgers’ Zoo*; vocal: *D* = 0.06, *p* = 0.241; *motoric*: *D* = 0.08, *p* = 0.319). This demonstrates that the rate of isochrony remains consistent despite variation between and within colonies and in the way in which the display is performed.

### (c) Individually specific tempi

**Figure 2.**
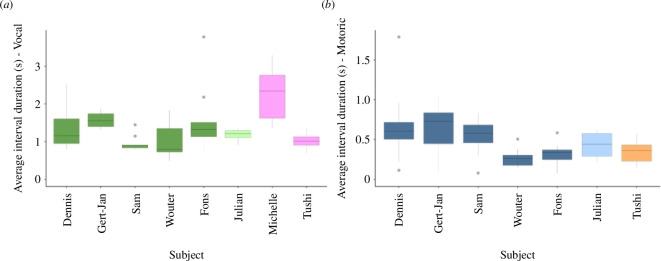
Visualization of the individual variation in the average duration between intervals (in seconds) per individual for both the vocal (*a*) and motoric (*b*) elements of display. This shows the individual differences in tempi of the display behaviour, with different colours that symbolize different demographic groups (adult males, juvenile males and adult females; vocal: dark green, light green and pink; motoric: dark blue, light blue and orange, respectively). For the results of statistical analysis, the variation in average interval duration between individuals was tested (electronic supplementary material, S6 and S7). Note that the figures have different *y*-axis scales because the inter-onset intervals within the motoric displays were, on average, longer than for the vocal displays.

The data indicated variation between the individuals, which had at least 25 interval ratios, vocally (figure 2*a*) and/or motorically (figure 2*b*). The individuals from different demographic groups (adult males, adult females and a juvenile male) exhibit varying temporal patterns in their displays (electronic supplementary material, S6 and S7). Specifically, the average duration of the intervals between the elements in a display sequence shows variation within and between individuals, creating individually distinct tempi in the isochronous behaviour. This results in some individuals generally displaying in a faster or slower tempo than other conspecifics. This variation does not seem to be correlated with sex, age, colony or group. Despite the difference in average interval duration, individuals perform their displays with high rates of isochrony. This indicates that there is no singular tempo at which this rhythmic behaviour is executed, either across different individuals or within the same individual. It also demonstrates that the displays are isochronous, regardless of the displaying individual or the tempo.

### (d) Context

**Figure 3.**
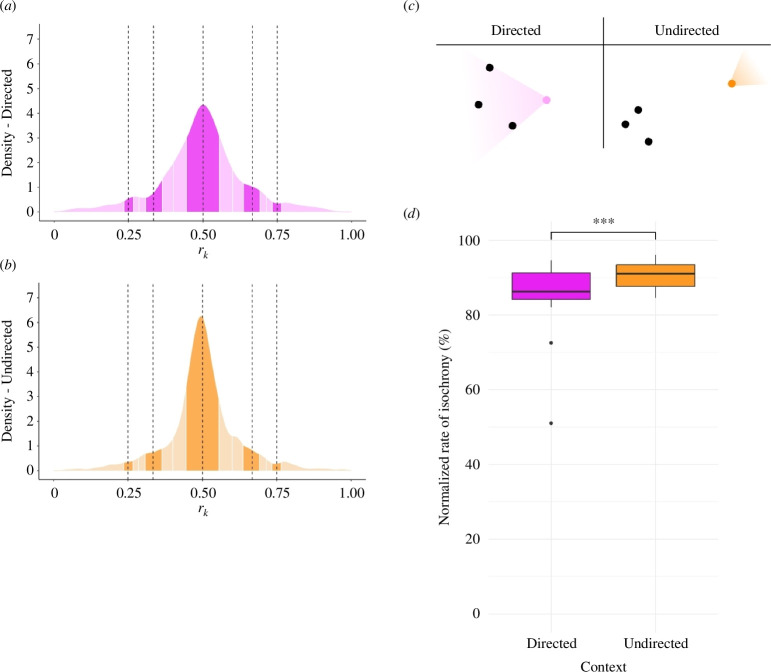
Visualization of the ratio distributions of directed displays ((*a*), directed at target individuals ((*c*)—left)) and undirected displays ((*b*), oriented toward the entire group ((*c*)—right)). The bins of the integer categorical rhythms are darker-shaded, with the neighbouring off-integer categories being lighter. Both contexts of display show a peak of interval ratios at isochrony. The undirected displays have a higher rate of isochrony than the displays in a directed context (electronic supplementary material, S4), which is depicted in a boxplot of the percentage of isochronous intervals, normalized by the size of the bin of this categorical rhythm (*d*).

Finally, the influence of the context in which the display behaviour occurred (directed: figure 3*a*, undirected: figure 3*b*) and its effect on interval variability and the rate of isochrony were analysed. Directed and undirected displays did not differ in interval variability (LMM, *coefficient of variation*; *directed* versus *undirected*: estimate = 0.035, s.e. = 0.038, *t* = 0.922, *p* = 0.357), but undirected displays were significantly more isochronous than directed displays (figure 3*a*,*b*; GLMM, *isochrony rate; directed* versus *undirected*: estimate = 0.524, s.e. = 0.153, *Z* = 3.428, *p* < 0.001). This lower rate of isochrony in the directed display was notably not correlated with, for instance, lower isochrony rate in vocal displays, because these were mostly undirected (interval ratios vocal display: *directed* = 503 and *undirected* = 1227). Apart from the significant difference in isochrony rate in both contexts, the interval ratios produced in either context were mainly isochronous.

## 4. Discussion

The aim of this study was to quantify the presence and properties of rhythm in the display behaviour of zoo-housed chimpanzees and the factors that could influence this rhythm. First and foremost, in the display behaviour, the individuals’ vocal and motoric sequences were both produced in isochronous rhythms. There was clear variation between modes of production, as motoric displays were more regular and had a higher isochrony rate than vocal displays. However, this variation could be (partly) owing to the different temporal precision with which the audio recordings of the vocal displays and the video recordings of the motoric displays could be analysed. Furthermore, there was variation in the duration of intervals, resulting in distinct individual display tempi. Nevertheless, overall, the display sequences were highly isochronous.

The clearest variation in the rate of isochrony was seen between the two display contexts, directed and undirected, with undirected displays having a higher isochrony rate than directed displays. It should be noted, however, that despite this difference, both contexts showed remarkably high isochrony rates. As undirected displays are not aimed at a target individual, displaying individuals are possibly more focused on themselves because external stressors are less prominent [32]. Directed displays result inevitably in a divided focus between the action of performing a display and the interaction with the targeted individual(s). One could imagine that charging behaviour, following reactive targets during the display, might shift the overall tempo of the behavioural sequence. This might explain why isochronous rhythms are maintained relatively less often in directed display sequences. An alternative hypothesis that is proposed in the literature is that isochrony and regularity in vocalizations or movements have a function in communication. For instance, Australian magpie-larks (*Grallina cyanoleuca*) respond more territorial to regular duets than to irregular duets, regardless of tempo [49], and female mice (*Mus musculus*) approach playbacks of regular sequences more than playbacks of irregular sequences [50]. However, these findings predict the opposite of what was found in the current study; in the chimpanzees, the undirected displays have higher isochrony rates than the directed displays. A study on wild chimpanzees showed that the frequency and structure of pant-hoot vocalizations during displays depended on the composition of the audience to whom these displays were directed [51]. In this study, all ‘directed’ displays were combined without considering the composition of the intended audience to whom these displays were directed. However, the audience composition might have affected the rhythmic regularity of the displays, and future studies should investigate this.

Factors such as demography and the specific behaviour performed in the display did not have a significant influence on the rate of isochrony in display sequences. Although adult males did display more often, as was also shown in previous research [27,28], they did not maintain a more consistent rhythm than individuals from other demographic groups. While the influence of these factors cannot fully be ruled out, our findings do suggest that there is a broad foundation for isochronous rhythm production in chimpanzee display behaviour. This rhythm does not seem to result from practice as the youngest individual contributing to the dataset was 3 years old, nor even to depend on the frequency of displays. Moreover, as individuals uniquely shape their display sequences, resulting in a broad behavioural repertoire [27,29–31], the consistent rate of isochrony across individuals in the four observed groups further signifies the universal base of this rhythmicity.

In the wild, groups of chimpanzees adjust the individual- and group-specific variation in the temporal structure of buttress drumming and vocalizations to guarantee intergroup and intragroup recognition [22,24,25]. Similarly, implementing variation has been reported in harbour seal pups (*Phoca vitulina)* that tweak their isochronous vocal sequences to produce a distinctly different call in the presence of conspecifics [14]. We found no difference in the regularity or rhythmic rate between the two observed colonies or the different housing groups within the zoos. This absence of intergroup variation could have been caused by an inability to produce a structural change in rhythm within the group. A more plausible explanation, however, is that there was no necessity for groups to create uniquely structured rhythms. The latter suggestion is supported by there being hardly any interaction between the two housing groups in Beekse Bergen. If no interaction occurs between the neighbouring groups, surely it is not essential for individuals to differentiate the group-specific rhythmic structure of a display. On top of that, the housing groups in Burgers’ Zoo were only recently separated (in appreciation of the birth of two young) and rotated between the inside and outside habitat of a single enclosure. Accordingly, individuals from these divided groups likely still recognized each other as members of the same collective group. The interval-tempi between individuals varied similarly to the previously observed interindividual variation in wild chimpanzee behaviour [22,24,25]. This variation confirms that display sequences are flexibly modulated in tempo while maintaining a consistently isochronous rhythm, both within and between individuals.

We aimed to quantify rhythmic patterns in both modes of production during chimpanzee display behaviour and found isochronous rhythms despite individual-specific structural variance in behavioural sequences. Since this (or similar) music-based method has been applied in researching rhythmic properties, categorical rhythms were recorded in the vocalizations of a few non-human primates [16,17,19]. We found that chimpanzees share these rhythmic patterns in vocal sequences and that this consistency also manifests itself in the motoric behaviour. These findings provoke additional questions regarding the concept of rhythmicity. Behavioural rhythms can arise from regular physiological oscillations (e.g. heartbeat), be a result of physical constraints (e.g. when a behaviour performed at maximum speed naturally becomes isochronous) or represent the most energy-efficient option. However, some rhythmic behaviours may emerge during higher cognitive processes, where rhythmicity might be generated with a certain intentionality. Although it is complex to differentiate these sources of isochrony, the nature of the behaviour in which rhythmic patterns are exhibited might offer clues as to whether it stems mainly from physical processes or it involves a cognitive component. For example, the difference between the isochrony rate of vocal and motoric behaviours of the chimpanzees might give some indication to the underlying processes. Assuming that physical restraints have a greater impact on movements than on vocalizations, this could potentially explain why the movements exhibit a more isochronous pattern. However, it should be noted that, as previously mentioned, the rhythmic differences between vocal and motoric behaviour could also be attributed to the different temporal resolutions of the analysis of audio and video recordings. Nevertheless, the isochrony in displays is likely an intricate interplay between physical constraints, energy efficiency and potentially an underlying cognitive mechanism.

Rhythmic patterns seem to be present in other social behaviours of chimpanzees and other non-human primates—for instance, lip-smacking behaviour that is performed in constant patterns, resembling human speech rhythm [52–55]. Our findings contribute to the expanding documentation that uses music-based methods to quantify rhythmic behaviours across species [12–17,19]. Consequently, the presence of rhythm, unaffected by variation between colonies, individuals, context and mode of production, further underlines the universality of this trait. This is in line with the general hypothesis that rhythm in human language and music evolved through a gradual process, with simple rhythmic capacities common across species and the advanced skills only present in a few [11]. Future research can investigate these shifts to advanced cognitive capacities, using these music-based methods in a broad range of species. This allows for a deeper understanding of how rhythm evolved to be the fundamental trait that it is for human language and music.

## Data Availability

The dataset and code for the statistical analysis used in this study has been uploaded as electronic supplementary material [56].
